# Alterations of the Oral Microbiota Profiles in Chinese Patient With Oral Cancer

**DOI:** 10.3389/fcimb.2021.780067

**Published:** 2021-12-09

**Authors:** Zixuan Li, Gang Chen, Panpan Wang, Minglei Sun, Junfang Zhao, Ang Li, Qiang Sun

**Affiliations:** ^1^ Department of Oral and Maxillofacial Surgery, First Affiliated Hospital of Zhengzhou University, Zhengzhou, China; ^2^ Shenzhen Stomatology Hospital (Pingshan), Southern Medical University, Shenzhen, China; ^3^ Health Management Center, The First Affiliated Hospital of Zhengzhou University, Zhengzhou, China

**Keywords:** oral cancer, oral microbiota, metagenomic sequencing, correlation, diagnostic value

## Abstract

Oral cancer is the most common malignant tumor in the oral and maxillofacial region, of which more than 90% is squamous cell carcinoma. The incidence of oral cancer is on the rise worldwide. An imbalance between the microorganism composition and its host may lead to the occurrence of oral malignant tumors. Accumulating evidence suggests that the oral microbiota plays an important role in oral cancer; however, the association between oral microbiota and oral cancer has not yet been comprehensively studied. In this study, metagenomic sequencing was used to compare the microbial composition of three groups of samples from Chinese patients with oral cancer, patients with precancerous lesion, and normal individuals. In terms of microbiota richness, the oral microbiota of patients with precancerous lesions was richer than that of oral cancer patients and healthy controls, whereas in terms of microbiota diversity, there was little difference between the three groups. The three groups of samples exhibited statistically significant differences in microbiota composition and metabolic function at the family, genus, and species levels (*P* < 0.05). The differentially enriched phylum in oral cancer samples was Bacteroidetes (*P* < 0.05). At the genus level, the main differentially enriched taxa were *Prevotella, Peptostreptococcus, Carnobacterium*, and *Diastella* (*P* < 0.05). The species level was differentially enriched in *Prevotella intermedia* and *Peptostreptococcus stomatis* (p < 0.05). The prediction of microbiota function shows that oral cancer is mainly associated with coenzyme A biosynthesis, phosphopantothenic acid biosynthesis, inosine 5’-phosphate degradation, and riboflavin biosynthesis. Furthermore, the increase in C-reactive protein level in oral cancer patients was found to be closely related to *P. intermedia*. Overall, oral bacterial profiles showed significant differences between the oral cancer group and normal group. Hence, microbes can be employed as diagnostic markers and treatment targets for oral cancer.

## Introduction

Oral cancer is the most common malignant tumor of the oral and maxillofacial region, and more than 90% of cases are squamous cell carcinomas (SCC) (Tandon et al., 2017). The incidence of oral cancer is increasing worldwide, and despite the remarkable progress in cancer treatment, the incidence and mortality rates of oral cancer remain high, with the 5-year survival rate staying at approximately 50% (Overman, 2006). Oral cancer mostly involves the whole tongue, larynx, jaws, important blood vessels, and functional nerves of the neck and skull base, as well as other important areas, which threatens the patient’s life and is accompanied by obvious dysfunction such as swallowing, speech, pain, numbness, and breathing problems, which greatly reduces the quality of life. The main risk factors of OSCC include smoking, betel nut chewing, and heavy alcohol consumption, but approximately 15% of oral squamous carcinomas cannot be explained by these main risk factors (Perera et al., 2018), and other potential risk factors need to be explored.

In the 1990s, researchers first demonstrated the pathogenic role of *Helicobacter pylori* in gastric cancer, linking carcinogenicity to the bacteria (Marwick, 1990). Subsequently, many studies evaluated the relationship between bacteria and cancers of other organs. For example, EBV was determined to be associated with Burkitt lymphoma and nasopharyngeal carcinoma (Allavena et al., 2008), an increased risk of gallbladder cancer was found to be associated with *Salmonella typhi* infection (Scanu et al., 2015), and HPV infection was linked to cervical cancer. These findings provide reference and new research directions for the relationship between microorganisms and oral tumors. Some studies have found (Rajeev et al., 2020) that oral microorganisms might be related to the development of oral tumors, but the mechanism is not clear. The research methods range from simple bacterial culture to PCR molecular techniques and next-generation sequencing-16S rRNA gene detection (Perera et al., 2016), and these have now progressed to metagenomic sequencing techniques. Microbial species variation associated with oral cancer is variable owing to differences in study results. Accumulating evidence suggests that the bacterial composition of the mucosal surface, inside tumor tissue, and in saliva of patients with oral squamous cell carcinoma is very different from that of the oral cavities of healthy individuals (Berkovits et al., 2016). The abundance of opportunistic pathogens in the saliva of oral cancer patients was found to be remarkably higher than that in healthy people (Pushalkar et al., 2011). Schmidt et al. (2014), using the 16SrDNA method, found that the abundance of *Streptococcus* and *Actinomycetes* in oral squamous cell carcinoma and precancerous lesions was significantly reduced, whereas the abundance of *Bacteroides* was significantly increased, indicating that the change in oral microbiota occurred in the early stage of cancer and that cancer progressed along with the development of the tumor. Yang et al. (2018) showed that the oral bacterial microbiota was different between healthy participants and patients with oral squamous cell carcinoma and that it changed during tumor progression. Yost et al. (2018) conducted a microbial macrotranscriptomic study on 15 samples and found that the microbial community in tumor sites of patients with oral squamous cell carcinoma changed significantly compared with that of the matching sites of healthy participants. Therefore, squamous cell carcinoma can be efficiently screened by detecting the oral microbiota. Changes in the oral microbial community structure reveal that these microorganisms might have a pivotal role in the prevention and early diagnosis of oral cancer.

Most studies now focus on tumor tissues and adjacent and contralateral normal tissues, but the distribution of microbiota in tissues is small and unstable. In our study, we collected samples of gargle fluid with relatively stable microbiota. The present study used metagenomic sequencing technology to detect the presence and distribution characteristics of oral microbiota in patients with oral cancer, precancerous lesions, and normal populations and explored the relationship between the occurrence of oral cancer and the oral microbial microbiota. This has implications for further mechanistic exploration and can be used as a biomarker to predict OSCC with high diagnostic accuracy.

## Materials and Methods

### Subject Enrollment

From February 2021 to April 2021, we collected 10 oral gargle samples from patients hospitalized in the First Affiliated Hospital of Zhengzhou University who were pathologically diagnosed with oral cancer, as well as from healthy participants; in addition, we collected samples from six patients with oral precancerous lesions. All patients provided an informed consent form and had complete clinical and pathological data. No other malignant tumors were found upon systemic examination, and distant metastases were excluded. Each participant was instructed to avoid smoking, drinking, and eating for at least 30 min before sample collection.

#### Inclusion Criteria

Patients were enrolled without severe periodontal disease, severe dental caries, or oral mucosal disease, with no other systemic diseases, no history of surgery, and no history of antibiotic application within 3 months before enrollment.

#### Exclusion Criteria

Patients were excluded with a history of oral infectious diseases or bleeding, history of antibiotics within 3 months before enrollment, having other history of other malignant tumors.

### Sample Collection, DNA Extraction, Metagenomic Sequencing, and Quantity Control of Reads

The gargle samples were rinsed with physiological saline three times and were then collected in a sterile 50 mL centrifuge tube, transported in an ice pack, and stored in an ultra-low temperature refrigerator at -80°C. DNA from a total of 26 samples was extracted using the MagPure Stool DNA KF kit according to the manufacturer’s instructions. DNA nanoball (DNB)-based DNA library construction and combinatorial probe-anchor synthesis (cPAS)-based shotgun metagenomic sequencing with 100 bp paired-end reads were applied to all samples (MGI2000, MGI, Shenzhen, China). Quality control (QC) of raw sequencing reads was applied to filter out low-quality reads using an overall accuracy (OA ≥ 0.8) control strategy as previously described (Fang et al., 2018). High-quality reads were aligned to hg19 using SOAPaligner/soap2 to filter out human reads (identity ≥ 0.9).

### Microbiome Composition Profiling

Taxonomic annotation and quantification was performed based on MetaPhlAn2 with default settings (Truong et al., 2015); the oral microbial profile was found to include bacteria, archaea, eukaryotes, and viruses. Additionally, taxon-specific community functional profiles were generated using HUMAnN2 (the HMP Unified Metabolic Analysis Network 2) (Franzosa et al., 2018).

### Assessment of Intermicrobial Interaction

We used Spearman’s correlation coefficient and microbiome relative abundance to identify intermicrobial interactions, i.e., interactions between pathways and species. We randomly subsampled 80% of all samples to bypass possible outliners and repeated this process 100 times; thereafter, the mean Spearman’s correlation coefficient and mean p-value were evaluated as the inner-interaction measurement.

### Calculation of Relative Abundance of Oral Microbiota

Metagenomic classification of sequenced libraries was carried out through Metaphlan2 to obtain standard relative abundance values of species at all levels. Starting from the 17,000 genomes available from the Integrated Microbial Genomes (IMG) system, we identified more than 2 million potential markers, from which we selected a subset of over 400,000 genes most representative of each taxonomic unit, and eventually, each species’ unique marker could be attend. Secondly, a comparison between the sequence and marker was performed. The MetaPhlAn classifier was used to compare metagenomic reads against this precomputed marker catalog *via* nucleotide BLAST searches in order to provide clade abundances for one or more sequenced metagenomes. Lastly, the content calculation was performed. The classifier normalized the total number of reads in each clade by the nucleotide length of its markers and provided the relative abundance of each taxonomic unit, accounting for any markers specific to subclades. The microbial clade anomaly was thus estimated by normalizing read-based counts by the average genome size of each clade.

### Statistical Analysis

Statistical analyses were performed using R program version 4.0.2. The analysis of differences between groups was performed by the rank sum test, and P ≤ 0.05 indicated statistical significance. Principal coordinate analysis (PCoA) was performed using the R program “ade4” according to the relative abundance of microbial species or pathways. We used the R program “vegan” package to calculate the Shannon index, Simpson index, observed species number (OBS), and gini index in each sample and performed a permutational multivariate analysis of variance (PERMANOVA) with the package “adonis2”. The R program “qvalue” was used to perform multiple tests. STAMP software (Parks et al., 2014) was used to determine the characteristic differences between the three groups of samples.

## Results

### Characteristics of the Primary Cohort

The final analysis and Metagenomic sequencing of the samples was carried out for 26 subjects, including the cancer, precancerous lesion, and control groups (n = 10, 6, and 10, respectively). The clinical characteristics of the study participants are listed in **Table 1**. The participants in the three groups exhibited no significant differences in terms of sex (*P* = 0.13), age (*P* = 0.38), and main demographic and socioeconomic characteristics, such as body mass indices (*P* > 0.05, for all). The demographic and laboratory test result data are summarized in **Table S1**.

**Table 1:** Clinical data of 26 cases of oral gargle samples.

Group	Gender	Age	BMI
Ca(n=10)	4F, 6M	59.4 ± 12.9	23.5
Normal(n=10)	5F, 5M	37.3 ± 11.2	22.1
Pre (n=6)	5F, 1M	56.5 ± 7.5	22.1
p value	0.13	0.38	0.20
F.Model	1.39	1.05	1.26

Ca, oral cancer group; Normal, the normal groups; Pre, precancerous lesion group.

F, female; M, male.

### Bacterial Populations and Core Microbiome in Three Groups of Oral Samples

In total, 282 species were obtained from the three sample groups, including 10 phyla, 20 classes, 26 orders, 36 families, and 75 genera. The dominant microbiota in the normal control, precancerous lesion, and oral cancer groups included Proteobacteria (19.26%) and Firmicutes (53.83%), Firmicutes (34.06%) and Bacteroides (29.03%), and Firmicutes (36.84%) and Bacteroides (33.91%), respectively. Furthermore, among the 36 families, 7 families, including *Corynebacteriaceae, Erysipelotrichaceae, Micrococcaceae, Peptostreptococcaceae, Prevotellaceae, Streptococcaceae* and *Carnobacteriaceae* were dominant (**Table 2**). Among the 75 genera, 9 genera, including *Corynebacterium, Dialister, Haemophilus, Peptostreptococcus, Prevotella, Rothia, Streptococcus, Granulicatella* and *Gemella* were dominant (**Table 3**).

**Table 2:** Abundances of dominant families with significant differences among the three groups as calculated using STAMP.

Phylum	Family	Ca	Normal	Pre	Enrich in	P
Actinobacteria	f_Corynebacteriaceae	0.08%	0.38%		Normal	0.002
Firmicutes	f_Erysipelotrichaceae	0.64%	0.17%		Ca	0.037
Actinobacteria	f_Micrococcaceae	2.60%	6.98%		Normal	0.005
Firmicutes	f_Peptostreptococcaceae	2.54%	0.50%		Ca	0.026
Bacteroidetes	f_Prevotellaceae	24.02%	9.69%		Ca	0.002
Firmicutes	f_Streptococcaceae	19.48%	39.83%		Normal	0.000
Firmicutes	f_Carnobacteriaceae	1.45%		0.63%	Ca	0.045
Firmicutes	f_Peptostreptococcaceae	2.53%		0.33%	Ca	0.017
Firmicutes	f_Bacillalesnoname		6.19%	2.08%	Normal	0.032
Firmicutes	f_Streptococcaceae		39.83%	21.52%	Normal	0.011

**Table 3:** Abundances of dominant genera with significant differences among the three groups as estimated using STAMP.

Phylum	Genus	Ca	Normal	Pre	Enrich in	P
Actinobacteria	g_Corynebacterium	0.01%	0.38%		Normal	0.002
Firmicutes	g_Dialister	0.37%	0.05%		Ca	0.039
Proteobacteria	g_Haemophilus	2.06%	4.56%		Normal	0.022
Firmicutes	g_Peptostreptococcus	1.60%	0.07%		Ca	0.039
Bacteroidetes	g_Prevotella	22.81%	8.64%		Ca	0.001
Actinobacteria	g_Rothia	2.60%	6.98%		Normal	0.005
Firmicutes	g_Streptococcus	19.47%	39.82%		Normal	0.000
Firmicutes	g_Dialister	0.37%		0.06%	Ca	0.042
Firmicutes	g_Granulicatella	1.41%		0.56%	Ca	0.036
Firmicutes	g_Peptostreptococcus	1.60%		0.07%	Ca	0.039
Firmicutes	g_Gemella		6.19%	2.08%	Normal	0.032
Firmicutes	g_Streptococcus		39.82%	21.52%	Normal	0.011

### Changes in Bacterial Composition Between the Three Groups

**Figure 1:**
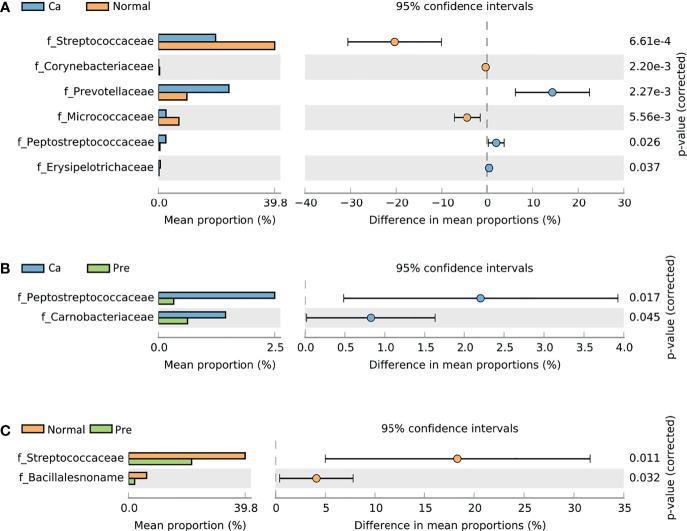
Comparative taxonomic profiles of the three groups at the family level showed significant differences in richness between two groups (P < 0.05, calculated using STAMP). **(A)** Difference between the oral cancer group and normal group. **(B)** Difference between the oral cancer group and precancerous lesion group. **(C)** Difference between the normal group and precancerous lesion group.

STAMP analysis revealed that the oral cancer and normal groups included the families Corynebacteriaceae (0.08%, 0.38%), Erysipelotrichaceae (0.64%, 0.17%), and Micrococcaceae (2.60%, 6.98%). Moreover, Peptostreptococcaceae (2.54%, 0.50%), Prevotellaceae (24.02%, 9.69%), and Streptococcaceae (19.48%, 39.83%) showed significantly different abundance (*p* < 0.05). Among these families, Corynebacteriaceae, Micrococcaceae, and Streptococcaceae were enriched in the normal group (**Figure 1A**), whereas Erysipelotrichaceae, Peptostreptococcaceae, and Prevotellaceae were mainly enriched in the oral cancer group. Furthermore, the oral cancer group and precancerous lesions of the two groups of samples revealed a statistically significant difference in Carnobacteriaceae (1.45%, 0.63%) and Peptostreptococcaceae (2.53%, 0.33%) (*p* < 0.05) abundance between the two groups; these families were mainly enriched in the oral cancer group (**Figure 1B**). In the normal group and the precancerous lesion group, the difference between the two groups in the abundance of Bacillalesnoname (6.19%, 2.08%) and Streptococcaceae (39.83%, 21.52%) was statistically significant (*p* < 0.05); these families were mainly enriched in the normal group (**Figure 1C**). The statistical data are summarized in **Table S2**.

**Figure 2:**
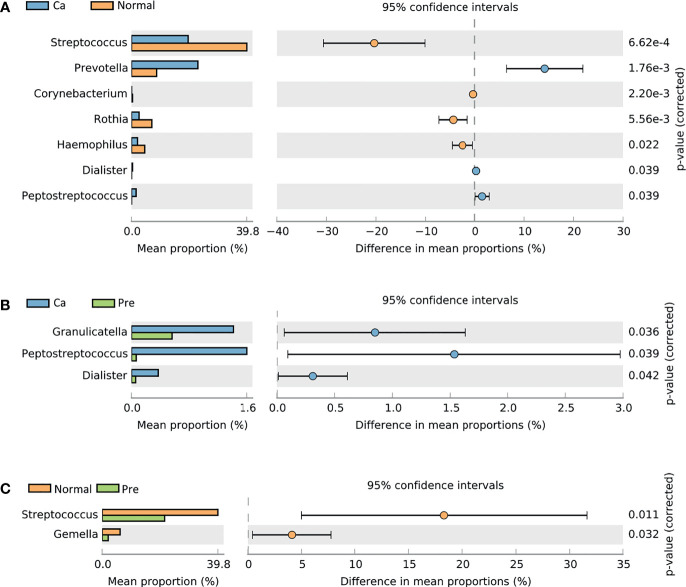
Comparison of taxonomic profiles at the genus level among the three groups showed significant differences in richness between two groups (P < 0.05, calculated using STAMP). **(A)** Difference between the oral cancer group and normal group. **(B)** Difference between the oral cancer group and precancerous lesion group. **(C)** Difference between the normal group and precancerous lesion group.

Additionally, in the oral cancer group and the normal group, the genera *Corynebacterium* (0.01%, 0.38%), *Dialister* (0.37%, 0.05%), *Haemophilus* (2.06%, 4.56%), *Peptostreptococcus* (1.60%, 0.07%), *Prevotella* (22.81%, 8.64%), *Rothia* (2.60%, 6.98%), and *Streptococcus* (19.47% 39.82%) revealed a statistically significant difference (*p* < 0.05); among these genera, *Dialister*, *Peptostreptococcus*, and *Prevotella* were enriched in the oral cancer group (**Figure 2A**), whereas *Corynebacterium*, *Haemophilus*, *Rothia*, and *Streptococcus* were enriched in the normal group. Moreover, *Dialister* (0.37%, 0.06%), *Granulicatella* (1.41%, 0.56%), and *Peptostreptococcus* (1.60%, 0.07%) significantly differed (*p* < 0.05) and were enriched in the oral cancer samples (**Figure 2B**), whereas *Gemella* (6.19%, 2.08%) and *Streptococcus* (39.82%, 21.52%) significantly differed (*p* < 0.05) and were enriched in the normal group (**Figure 2C**). The statistical data are summarized in **Table S3**.

**Figure 3:**
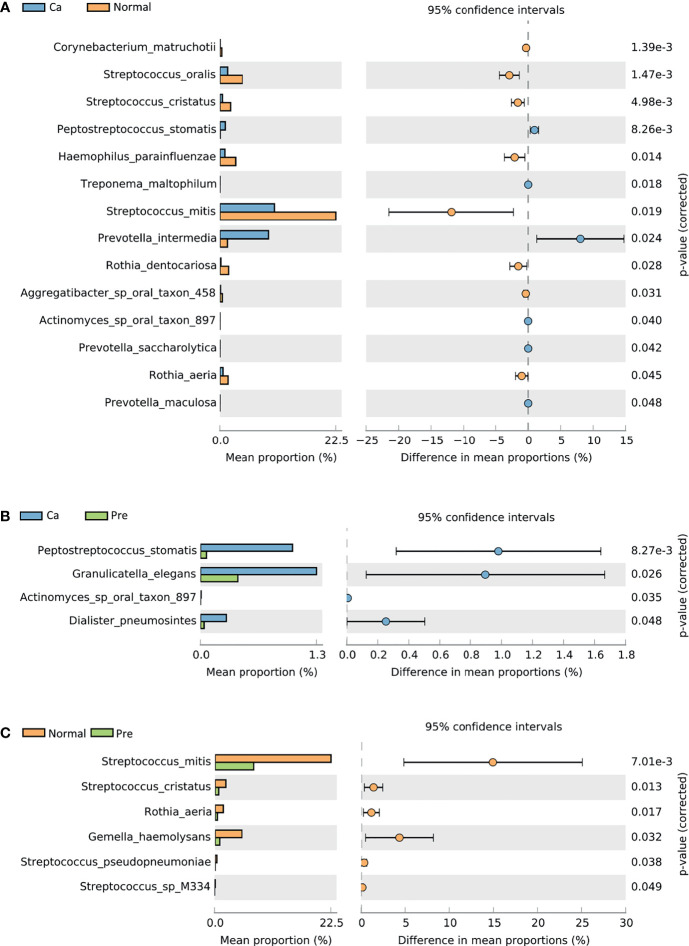
Comparative taxonomic profiles at the species level showed significant differences in richness between two groups (P < 0.05, calculated using STAMP). **(A)** Difference between the oral cancer group and normal group. **(B)** Difference between the oral cancer group and precancerous lesion group. **(C)** Difference between the normal group and precancerous lesion group.

STAMP was used to identify 282 species with abundant differences in the group. In the oral cancer group and the normal groups, fourteen species, including *P. intermedia* (9.51%, 1.47%) and *Peptostreptococcus stomatis* (1.05%, 0.07%), revealed a statistically significant difference (*p* < 0.05); however, in the normal group, *Haemophilus parainfluenzae* (0.98%, 3.05%), *Corynebacterium matruchotii* (0.05%, 0.36%), *Rothia aeria* (0.19%, 1.69%), *Streptococcus mitis* (10.64%, 22.52%), and *Streptococcus oralis* (1.51% 4.40%) were enriched (**Figure 3A**). In the oral cancer group and precancerous lesion group, four species, including *Actinomyces* sp oral taxon 897 (0.01%, 0.00%), *Dialister pneumosintes* (0.29%, 0.04%), *Granulicatella elegans* (1.32%, 0.42%), and *P. stomatis* (1.05%, 0.07%), exhibited a statistically significant difference (*p* < 0.05) (**Figure 3B**). In the normal group and the precancerous lesion groups, six species, namely *Gemella haemolysans* (5.21%, 0.89%), *Rothia aeria* (1.58%, 0.44%),*Streptococcus cristatus* (2.08%, 0.69%), *Streptococcus mitis* (22.51%, 7.57%), *Streptococcus pseudopneumoniae* (0.33%, 0.01%), and *Streptococcus* sp M334 (0.15%, 0.00%) exhibited a statistically significant difference (*p* < 0.05) (**Figure 3C**). The statistical data are summarized in **Table S4**.

**Figure 4:**
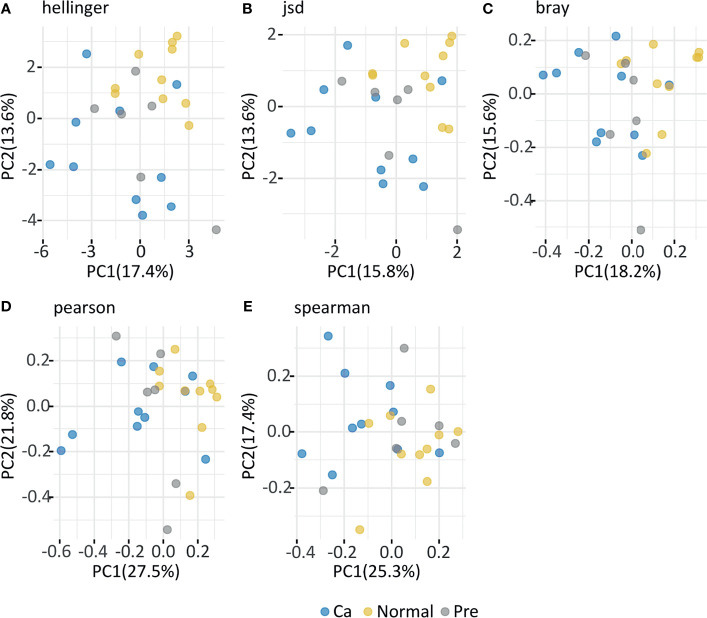
In the figure depicting Principal co-ordinates Analysis (PCoA), blue dots represent a gargle sample from the oral cancer group, yellow dots represent a gargle sample from the normal group, and gray dots represent a gargle sample from the precancerous group. A dot represents a sample, with similar samples arranged closer to each other. **(A)** Hellinger index; **(B)** Jsd index; **(C)** Bray index; **(D)** Pearson index; **(E)** Spearman index.

The normal group was similar to the precancerous lesion group. In contrast, the β diversity of the oral cancer sample group markedly differed from that of the other two groups. In the Hellinger index (**Figure 4A**), principal components 1 and 2 accounted for 17.4% and 13.6% of the differences, respectively, and the result of the Kruskal–Wallis test revealed no significant difference (*p* = 0.10) between groups. In the Jsd index (**Figure 4B**), principal components 1 and 2 accounted for 15.8% and 13.6% of the difference, respectively, and the Kruskal–Wallis test indicated a significant difference between the two groups (*p* = 0.04). In the Bray index (**Figure 4C**), principal components 1 and 2 accounted for 18.2% and 15.6% of the difference, respectively, and the Kruskal–Wallis test indicated a significant difference between the two groups (*p* = 0.04). In the Pearson index (**Figure 4D**), principal components 1 and 2 accounted for 27.5% and 21.8% of the difference, respectively, and the Kruskal–Wallis test indicated a significant difference between the two groups (*p* = 0.01). In the Spearman index (**Figure 4E**), principal components 1 and 2 accounted for 25.3% and 17.4% of the difference, respectively, and the Kruskal–Wallis test indicated a significant difference between the two groups (*p* = 0.03). The β diversity analysis revealed that individuals in the normal and the precancerous lesion groups were mixed together, whereas the oral cancer group samples were specific and different from the other two groups.

**Figure 5:**
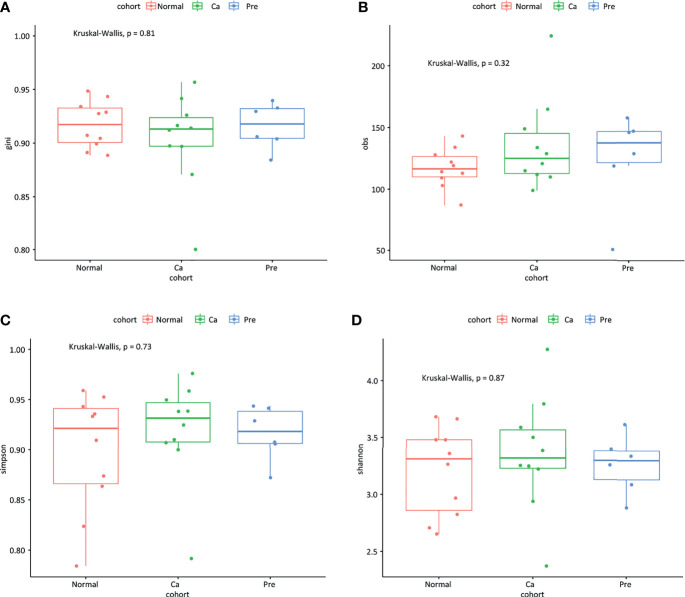
The temporal dynamics of microbiota alpha diversity; **(A)** gini index; **(B)** obs index; **(C)** Simpson index; **(D)** Shannon index.

In terms of microbiota richness, we found that the oral microbiota of patients with precancerous lesions was richer compared with that of oral cancer patients and healthy controls (gini index and obs index; **Figures 5A, B**); however, the difference was not statistically significant (P = 0.81, P = 0.32). In terms of microbiota diversity, there was a small difference between the three groups (Simpson index and Shannon index; **Figures 5C, D**) (*p* = 0.73, *p* = 0.87).

### Analysis of Differences Between Samples at Different Classification Levels

**Figure 6:**
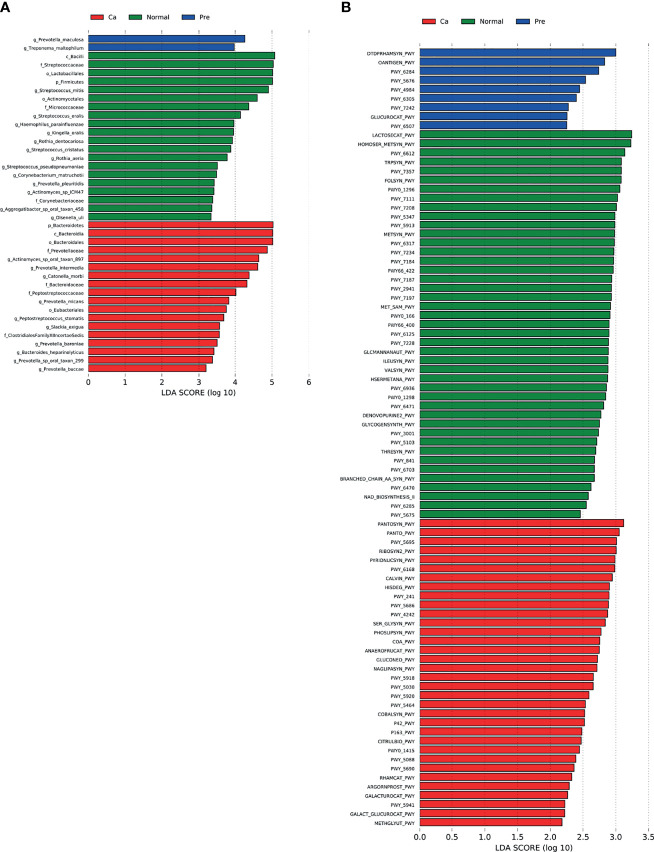
Analysis of markers between samples; green represents the normal control group, red represents the oral cancer group, and blue represents the precancerous lesion group. lgLDA >2 is significant. **(A)** The LEfSe algorithm was used to analyze the bacteria in different groups. **(B)** The LEfSe algorithm was used to analyze the function of bacteria in different groups.

As illustrated in **Figure 6A**, the significant differences in the oral cancer group were mainly clustered in the phylum Bacteroides and at genus *Prevotella*, those in healthy participants were mainly concentrated in Firmicutes and Streptococcus, and those in the precancerous lesion group was mainly concentrated in *Prevotella* and *Treponema*.

### Predicted Functional Changes in the Microbiomes of the Three Groups

Based on the selection of LDA, a search in the METACYC database revealed that in the oral cancer group, the function of the predicted microorganisms markedly increased in coenzyme A biosynthesis, phosphopantothenic acid biosynthesis, inosine 5’-phosphate degradation, and riboflavin biosynthesis compared to the healthy control group. In the healthy population, the function of the predicted 43 microorganisms markedly increased in lactose and galactose degradation; biosynthesis of L-methionine, tetrahydrofolate, and L-tryptophan; and formation of thiamine phosphate. In the precancerous lesion group, this function markedly increased in biosynthesis of dTDP-β-L-rhamnose, O-antigen, and unsaturated fatty acid as well as nine other factors (**Figure 6B**). The statistical data are summarized in **Table S5**.

### Diagnostic Model of Disease Prevalence

**Figure 7:**
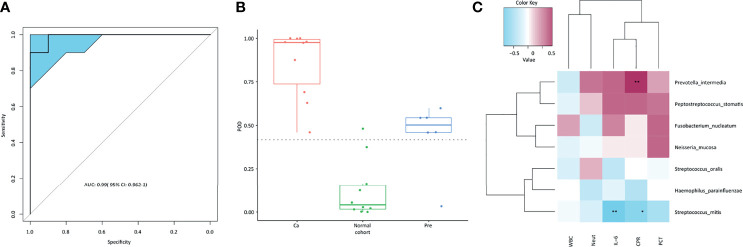
**(A)** ROC curve; **(B)** POD model; **(C)** relative analysis of microbiota and inflammatory indexes, the two asterisks marked in the figure indicate significance at a level of < 0.05; one asterisk indicates 0.05 < significance level < 0.1, and those not marked are considered not significantly related.

Three variables, *P.stomatis*, PWY_5103, and PWY_7242, were selected as models. The probability of illness for each sample was determined (**Figure 7B**). As illustrated in **Figure 7B**, the incidence of cancer in the cancer group was significantly higher than that in the normal control group, whereas the incidence of precancerous lesions in the group was slightly higher than that in the Cutoff value, with a cutoff value of 0.4175. The ROC curve (**Figure 7A**) was used to calculate the AUC value (AUC = 0.99) and 95% confidence interval (CI, 0.962–1).

### Correlation Analysis of Bacteria and Inflammatory Indexes in Patients With Oral Cancer

By analyzing the correlation between the microbiota and inflammatory indexes in patients with oral cancer (**Figure 7C**), we found that *P.intermedia* was positively correlated with procalcitonin (PCT), C-reactive protein (CPR), IL-6, and The neutrophil ratio (Nent), while *S.mitis* was negatively correlated with procalcitonin (PCT), C-reactive protein (CPR), IL-6, The neutrophil ratio (Nent), and white blood cells (WBCs).

## Discussion

The oral microbiome is one of the most complex microbial communities in the human body, with the oral cavity comprising more than 700 microorganisms (Vogtmann et al., 2017). Ecological imbalances in microbial communities, or ecological dysbiosis, have been extensively studied in human and animal models (Meurman et al., 2009). These are characterized by the loss of beneficial microorganisms, expansion of pathogenic microorganisms, and a general loss of microbial diversity (Xuan et al., 2014). It is becoming increasingly evident that ecological dysbiosis might contribute to cancer development (Schwabe and Jobin, 2013). Various beneficial or pathogenic microorganisms colonize the oral cavity, eventually leading to good health or diseases (Lamont et al., 2018). Previous studies (Fan et al., 2018; Ren et al., 2019; Ren et al., 2020) have verified the relationship between bacteria and cancer in various parts of the body, including the oral cavity. In recent years, researchers (Sheflin et al., 2014) have found that the role of a single species is transferred to the role of the microbial community. The oral cavity includes the hard teeth and the soft mucosal epithelium, making the oral environment relatively complex. In general, 50–1,000 different microbial species are simultaneously present in the oral cavity, that is, in the saliva, gingival sulcus, dental plaque, and tongue (Dewhirst et al., 2010). Chronic and persistent inflammation is a leading cause of cancer (Rewell, 1965). Previous studies have also reported an association between bacterial infection and carcinogenesis (Helicobacter et al., 2001; Littman, 2005). *H. pylori* plays a pivotal role in the occurrence and progression of gastric cancer (Kim et al., 2011; Moss, 2017). Furthermore, microbes and their products such as endotoxin (lipopolysaccharide), enzymes (protease, collagenase, fibrinolysis enzyme, and phospholipase A), and metabolic byproducts (hydrogen sulfide, ammonia, and fatty acids) exhibit toxicity to their surrounding cells and might directly induce mutations in tumor suppressor genes and proto-oncogenes or alter the signaling pathways associated with epithelial cell proliferation. Exogenesis can also be stimulated by indirect mechanisms that activate the peripheral inflammatory cells, exposing epithelial cells to a mutagenic microenvironment. Herein, microbes and their products were found to activate granulocytes, macrophages, monocytes, lymphocytes, fibroblasts, and epithelial cells to produce reactive oxygen species (hydrogen peroxide and oxygen free radicals), reactive nitrogen species (nitric oxide), lipid metabolites, and matrix metalloproteinases. These substances can induce DNA damage in epithelial cells and might also lead to the production of cytokines, chemokines, growth factors, and other signals for cell survival, proliferation, migration, angiogenesis, and apoptosis inhibition (Karin et al., 2006). The microbial community and the host mostly maintain a dynamic balance; some beneficial bacteria can effectively counter the invasion of external pathogens and enhance the tissues and immune system. Moreover, poor eating habits, lifestyle, and a weak immune system might lead to imbalances and could cause the overgrowth of pathogenic bacteria, thereby triggering various oral diseases, such as tooth decay, periodontal disease, oral leukoplakia, compressed moss, and oral cancer (Suwannakul et al., 2010; Valm et al., 2011).

In the present study, we aimed to determine the relationship between oral microbiota characteristics and oral squamous cell carcinoma. Through our analysis, we found significant changes in the oral microbiota among the three groups. In terms of the oral microbial composition, Actinobacteria, Firmicutes, Bacteroidetes, and Proteobacteria, were the four main phyla in the oral cavity. At the family level, Erysipelotrichaceae, Peptostreptococcaceae, Prevotellaceae, and Carnobacteriaceae were mainly enriched in the oral cancer group. Among these, the genera *Dialister*, *Peptostreptococcus*, *Prevotella*, and *Granulicatella* were enriched in the oral cancer group. Further, *S. cristatus*, *G. elegans*, *P. intermedia*, and *P. stomatis* were significantly increased in abundance at the species level. These are present in the oral mucosa as commensal bacteria but might be opportunistic pathogens of potential relevance in relation to oral squamous cell carcinoma. Simultaneously, the precancerous lesion group was included in this study to represent a transition between the oral cancer group and the healthy control group.

*Fusobacterium nucleatum*, a gram-negative bacillus, is one of the important pathogenic bacteria of periodontal disease. It was found that infection with periodontal pathogens and the development of oral cancer are closely related (Tezal et al., 2007). *F. nucleatum* is widely found in the gastrointestinal tract as an oral colonizing bacterium and has been shown to be virulent or pathogenic and an opportunistic pathogen (Abe et al., 2011; Brennan et al., 2018). In the present study, the abundance of *F. nucleatum* increased sequentially in the normal (6.48%), precancerous (15.07%), and cancerous (40.88%) groups. This is in agreement with the findings of Ganly et al. (2019) in 2019. Meanwhile, one study detected *F. nucleatum* in the saliva, dental plaque, oral exfoliated cells, cancerous tissues, and paracancerous tissues of patients with oral squamous carcinoma by quantitative PCR and 16S rRNA, with the highest abundance, and manifestation as an inflammatory microorganism, in cancerous tissues (Al-hebshi et al., 2017). *F. nucleatum* can also promote the progression of oral squamous carcinoma and is associated with poor prognosis with this disease. (Yang et al., 2018; Zhang et al., 2019) *F. nucleatum* is an oral symbiotic bacterium (Brennan et al., 2018), which has the ability to colonize, adhere to, and invade tissues, promote the proliferation and migration of host and tumor cells, and also interact with other microbiota, thus affecting the body’s immune function and promoting the formation of inflammatory and tumor microenvironments (Kostic et al., 2013; Nosho, 2016).

*P. intermedia* and *P. stomatis* were relatively abundant in the oral cancer groups. *Prevotella* is closely associated with chronic inflammatory diseases and is present in biofilms of gingivitis and periodontal disease (Dahlen, 1993; Berezow et al., 2011). The mechanism involves neutrophil recruitment and the expression of proinflammatory cytokines and metalloproteinases that mediate the destruction of connective tissue and alveolar bone. Presently, most studies on this mechanism have focused on the role of *Porphyromonas gingivalis* (a gram-negative anaerobic member of Bacteroides) (Hajishengallis, 2014). *P. stomatis* is a gram-positive anaerobic bacterium and an obligate parasite of the oral cavity, mucous membrane, and intestinal tract of mammals; it might also play a pivotal role in suppurative infection and is closely related to the occurrence of periodontal disease (Rams et al., 1992). Previous studies have reported that chronic periodontal disease is associated with the development of head and neck cancer, particularly oral squamous cell carcinoma. For every millimeter of alveolar bone loss, the risk of head and neck squamous cell carcinoma increases four-fold. In addition, those with periodontal disease are at a higher risk of developing poorly differentiated cancers (Tezal et al., 2009; Javed et al., 2016). As periodontal disease is a type of microbial disease, it provides a foundation for studying the etiology of oral cancer with respect to microorganisms.

We should also not ignore taxa with low abundances but significantly increased in OSCC, as these taxa could be considered “key” microorganisms and might be more virulent and therefore have an increased role in the development of cancer. *G. elegans* is a fastidious gram-positive coccus (pairs, chains) and is part of the nutritionally variant streptococci (NVS) group. Like other constituents of the NVS, it can cause bacteremia and infective endocarditis, which are associated with significant morbidity and mortality. *D. pneumosintes* (formerly named *Bacteroides pneumosintes*) is a non-fermentative, anaerobic, gram-negative rod. *D. pneumosintes* has been recovered from deep periodontal pockets, but little is known about the relationship between this organism and destructive periodontal disease (Doan et al., 2000).

The present study revealed that at the phylum level, the relative abundance of Bacteroidetes in the oral cancer and precancerous lesion groups was markedly higher than that in healthy subjects. Schmidt et al. (2014) used the 16S rDNA method and found that the abundance of Streptococcus and Actinomycetes in oral squamous cell carcinoma and precancerous lesions was markedly reduced, whereas that of Bacteroides was remarkably increased, thereby indicating that the change in oral microbiota occurred during the early stage of cancer and might indicate the progression of cancer along with tumor development. This finding is consistent with the results of the present study. Yost et al. (2018) conducted a microbial macrotranscriptome study on 15 samples and found that compared with that of the matching sites of healthy participants, the microbial community in tumor sites of oral squamous cell carcinoma patients changed remarkably; *Streptococcus* and *Haemophilus* were highly abundant in the healthy control group, which was also consistent with the results of this study.

There is a wide variety of inflammatory biomarkers, which are of great importance for the diagnosis and treatment of clinical diseases, as well as for prognosis. The neutrophil ratio, C-reactive protein (CRP), procalcitonin (PCT), white blood cells (WBCs), and IL-6 have been extensively studied as markers of long-term prognosis in many cancers. Most studies have demonstrated that the postoperative survival of cancer patients correlates with the systemic inflammatory response (Ethier et al., 2017). In the early stages of tumor proliferation and metastasis, systemic inflammatory markers can signify pro-cancer inflammation in the tumor microenvironment (Choi et al., 2016). Multiple cellular interactions with inflammatory factors further promote tumor formation due to an extremely immunosuppressive microenvironment (Kummar et al., 2007). Neutrophils play an essential role as they exert various potential effects in cancer, including promoting metastasis. In the inflammatory response to cancer, neutrophils can act directly with circulating tumor cells, acting as reservoirs of circulating vascular endothelial growth factors and promoting metastasis. In addition, the metabolites of neutrophils, such as reactive oxygen species and proteases, have been shown to have clear and specific roles in regulating tumor cell proliferation, angiogenesis, and metastasis. Recently it has been shown that neutrophils can have cytotoxic effects on tumor cells (Jensen et al., 2009). Elevated CRP is a marker of systemic inflammation and is considered a predictor of poor survival in patients with various cancers (Crumley et al., 2006). PCT is an inactive pro-peptide substance of serum calcitonin and is barely detectable in blood under healthy physiological conditions (<0.1 ng/mL). Some studies have shown that PCT is significantly increased with fungal and virus infections (Clementi et al., 2017). IL-6 promotes cancer development by activating endothelial cells, increasing adhesion molecule expression, and inducing matrix metalloproteinase production. In conclusion, there is a close relationship between malignancy and inflammatory markers.

In this study, the MATECYC database was used to retrieve results. Coenzyme A biosynthesis, phosphopantothenic acid biosynthesis, inosine 5’-phosphate degradation, and riboflavin biosynthesis participate in various metabolic activities in the body, such as DNA synthesis, respiration, and oxidative metabolism. These metabolic activities are related to cell proliferation and might further lead to the development of tumors. The metabolic pathways of the normal control group included lactose and galactose degradation, L-methionine biosynthesis, tetrahydrofolate biosynthesis, L-tryptophan biosynthesis, and thiamine phosphate formation, wherein the oral cancer group and the healthy control group revealed significant differences in the metabolic pathways between related microbiota. The decrease in the phosphotransferase system and glycolysis and galactose metabolism might reflect a community response to reduced sugar sources on the tumor surface, as increased glucose uptake is critical for OSCC cell survival (Eckert et al., 2018). Notably, the function of O-antigen biosynthesis in microorganisms was increased in precancerous lesion samples. Lipopolysaccharide is composed of fat and polysaccharides containing an O-antigen, outer core, and inner core connected by covalent bonds. Therefore, whether the increase in the microbial O-antigen biosynthesis function is related to an increase in lipopolysaccharide biosynthesis remains to be elucidated.

Previously, major research on oral disease microorganisms was limited to simple bacterial culture technology. Owing to the limitations of bacterial culture, some microorganisms are difficult or impossible to cultivate. Metagenomic sequencing technology has the advantages of unbiasedness, wide coverage, and rapid detection. Research on the abundance and diversity of key bacterial groups through metagenomic sequencing can increase the clinical diagnostic value of oral malignancy. The diagnostic value of tumors reveals the potential of microbial markers as a noninvasive diagnostic tool for oral malignancies, which is conducive to the early diagnosis and treatment of oral malignancies. The limitation of this study is that the sample size was small. In future research, we will verify the diagnostic potential of oral microbial microbiota changes in oral malignant tumors using more samples.

## Conclusion

In summary, we have found significant structural changes in the oral microbiota of patients with oral cancer, patients with precancerous lesions, and healthy controls at different species levels. Our research showed that dysbiosis of the oral microbiota changes certain metabolic pathways to affect oral health. Future studies are still warranted to investigate the correlation between oral microbiota imbalances and oral cancer, providing new evidence for microbiota-targeted approaches for disease prevention.

## Data Availability Statement

The original contributions presented in the study are included in the article/**Supplementary Material**. Further inquiries can be directed to the corresponding author. The data that support the findings of this study have been deposited into CNGB Sequence Archive (CNSA) of China National GeneBank DataBase (CNGBdb) with accession number CNP0002274 http://db.cngb.org/cnsa/project/CNP0002274/reviewlink/.

## Ethics Statement

This study was approved by the Ethics Committee from the First Affiliated Hospital of Zhengzhou University (KY-2019-LW007). Informed consent was obtained from all participants.

## Author Contributions

ZL and QS conceived and designed the experiments. ZL, GC, PW, JF, MS, and AL performed the experiments. AL and ZL analyzed the data. ZL wrote the paper and edited the manuscript. The final manuscript was read and approved by all authors.

## Funding

This present work was funded by the grants of the Henan Natural Science Foundation project (21300410391), Henan Province Young and Middle-aged Health Science and Technology Innovative Talents (Outstanding Youth) Project (YXKC2020030), Henan Province Medical Science and Technology Research (Provincial and Ministry Joint Construction Key) Project (SBGJ202002071), Henan Medical Science and Technology Research Program (JointLy Built, LHGJ20200270), and National Natural Science Foundation of China Youth Fund (81702979). Henan Provincial Science and Technology Department Key Research and Development and Promotion Special (212102310592), Overseas Talent Program of Henan Provincial Health Care Commission (2018048). 

## Conflict of Interest

The authors declare that the research was conducted in the absence of any commercial or financial relationships that could be construed as a potential conflict of interest.

## Publisher’s Note

All claims expressed in this article are solely those of the authors and do not necessarily represent those of their affiliated organizations, or those of the publisher, the editors and the reviewers. Any product that may be evaluated in this article, or claim that may be made by its manufacturer, is not guaranteed or endorsed by the publisher.

## Supplementary Material

Supplementary Table 1 The demographic and laboratory test result data.

Supplementary Table 2 Statistical data on microbiota at the family level.

Supplementary Table 3 Statistical data on microbiota at the genus level.

Supplementary Table 4 Statistical data on microbiota at the species level.

Supplementary Table 5 Microbial pathways associated with three groups.
